# Metabolic Overlap in Environmentally Diverse Microbial Communities

**DOI:** 10.3389/fgene.2019.00989

**Published:** 2019-10-17

**Authors:** Eric R. Hester, Mike S. M. Jetten, Cornelia U. Welte, Sebastian Lücker

**Affiliations:** Department of Microbiology, Radboud University, Nijmegen, Netherlands

**Keywords:** bioinformatics, metagenomics, microbial communities, metagenome assembled genomes (MAGs), niche, functional redundancy

## Abstract

The majority of microbial communities consist of hundreds to thousands of species, creating a massive network of organisms competing for available resources within an ecosystem. In natural microbial communities, it appears that many microbial species have highly redundant metabolisms and seemingly are capable of utilizing the same substrates. This is paradoxical, as theory indicates that species requiring a common resource should outcompete one another. To better understand why microbial species can coexist, we developed metabolic overlap (MO) as a new metric to survey the functional redundancy of microbial communities at the genome scale across a wide variety of ecosystems. Using metagenome-assembled genomes, we surveyed nearly 1,000 studies across nine ecosystem types. We found the highest MO in extreme (i.e., low pH/high temperature) and aquatic environments, while the lowest MO was observed in communities associated with animal hosts, the built/engineered environment, and soil. In addition, different metabolism subcategories were explored for their degree of MO. For instance, overlap in nitrogen metabolism was among the lowest in animal and engineered ecosystems, while species from the built environment had the highest overlap. Together, we present a metric that utilizes whole genome information to explore overlapping niches of microbes. This provides a detailed picture of potential metabolic competition and cooperation between species present in an ecosystem, indicates the main substrate types sustaining the community, and serves as a valuable tool to generate hypotheses for future research.

## Introduction

Microorganisms drive global biogeochemical cycles, but they do not work or live in isolation. In order for any living species to survive, they must engage in competition for space and resources with other organisms that share similar nutritional requirements. The concept of loss of species less adapted relative to their competitors is known as competitive exclusion (Gause, 1934). When one species cannot sufficiently persist in a habitat, they become locally extinct. Through selection of traits that reduce the dependence on a common resource, populations may shift toward coexistence. This is known as niche partitioning, whereby competition is avoided through the utilization of different resources (Schoener, 1974). Evidence that these ecological and evolutionary forces shape microbial communities is prevalent in literature; however, the strength of these forces varies with the availability of resources [reviewed in (Nemergut et al., 2013)].

Describing a niche of an organism has remained challenging ever since the concept first emerged (Hutchinson, 1957). Typically, closely related species are thought to share similar niches, assuming their evolutionary relatedness is reflected in their nutritional requirements (Langille et al., 2013). Recently, neutral genetic markers have emerged as a proxy to measure species’ divergence on an evolutionary timescale; however, these phylogenetic markers (i.e., 16S rRNA genes) are unsuitable to evaluate differences in the biochemical capacity of the organisms (Caro-Quintero and Konstantinidis, 2012). Whole genomes contain information relevant to the metabolic capacity of a species, which is essential to describe the putative niches a microbial species may occupy. If one were to ask about the overlap of two microorganisms’ niches, it is conceivable that this is akin to asking how similar the two are on a genomic level. Consequently, the metabolic niche of an organism can be predicted from the genome. However, the metabolic niche must be distinguished from the fundamental niche, which includes factors such as morphological features or transcriptional and translational regulation. These features also influence an organism’s adaptation and persistence in a community, but their inclusion introduces additional complexities that are largely absent from genomics-based investigations.

With the continued advancement in high-throughput DNA sequencing, large amounts of genomic data are frequently released and available for public use. Several recent publications have reported thousands of novel bacterial and archaeal metagenome-assembled genomes (MAGs; Anantharaman et al., 2016; Parks et al., 2017; Delmont et al., 2018; Tully et al., 2018). The sequencing data originated from hundreds of studies investigating different ecosystems, such that these genomes represent a diverse set of taxa from ecosystems around the globe. This presents an opportunity to address the following important questions: how variable is niche overlap in microbial communities across different ecosystems and does the nature of the overlap (i.e., abundance of genes involved in nitrogen cycling) change based on habitat?

In the current study, we surveyed niche overlap in microbial communities by searching for shared pathways in the metabolic reaction network of species within these communities, which we refer to as “metabolic overlap” (MO). This approach was used to investigate two main questions. First, does the degree of niche overlap in microbial communities vary between ecosystems (i.e., do some communities have more species that utilize the same substrates)? Second, how do these microbial communities vary in the degree of overlap of different metabolic categories (e.g., nitrogen or sulfur metabolism)?

We observed patterns of overlap in microbial community members’ metabolism across different ecosystems, which were largely consistent with literature reports (Martiny et al., 2006; Kelly et al., 2014; Reese et al., 2018). For instance, a low degree of MO was found in microorganisms involved in highly specialized animal host–microbe associations, while aquatic microbes displayed a cosmopolitan repertoire of strategies for nutrient acquisition. These variations seem to be driven by different categories of metabolism, depending on the ecosystem. In addition, we addressed the question of how much the phylogenetic relationship of microbes corresponds to their MO. We found that phylogenetic distance between microorganisms was indeed a good predictor for the degree of MO. The strength of this relationship, however, varied between different ecosystems. Generally, survey-based metrics like MO enable observations of global trends and prompt fundamental questions about the biology and ecology of microorganisms.

## Results

### Definition of MO

**Figure 1:**
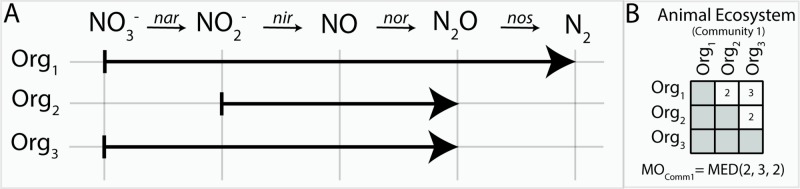
Metabolic overlap is a metric that compares the overlap in the metabolism of two organisms by calculating the number of reactants these species can utilize in common. This is determined by establishing their shared biochemical pathways **(A)** and counting which reactants both can use in common (i.e., common reactants utilized by organisms 1 and 2 is NO_2_^−^ and NO; thus, the MO_org(1,2)_ = 2). The number of substrates shared between a set of organisms is represented in a matrix **(B)**. Once all pairwise MO comparisons have been made for a community, the median metabolic overlap can be calculated.

**Figure 2:**
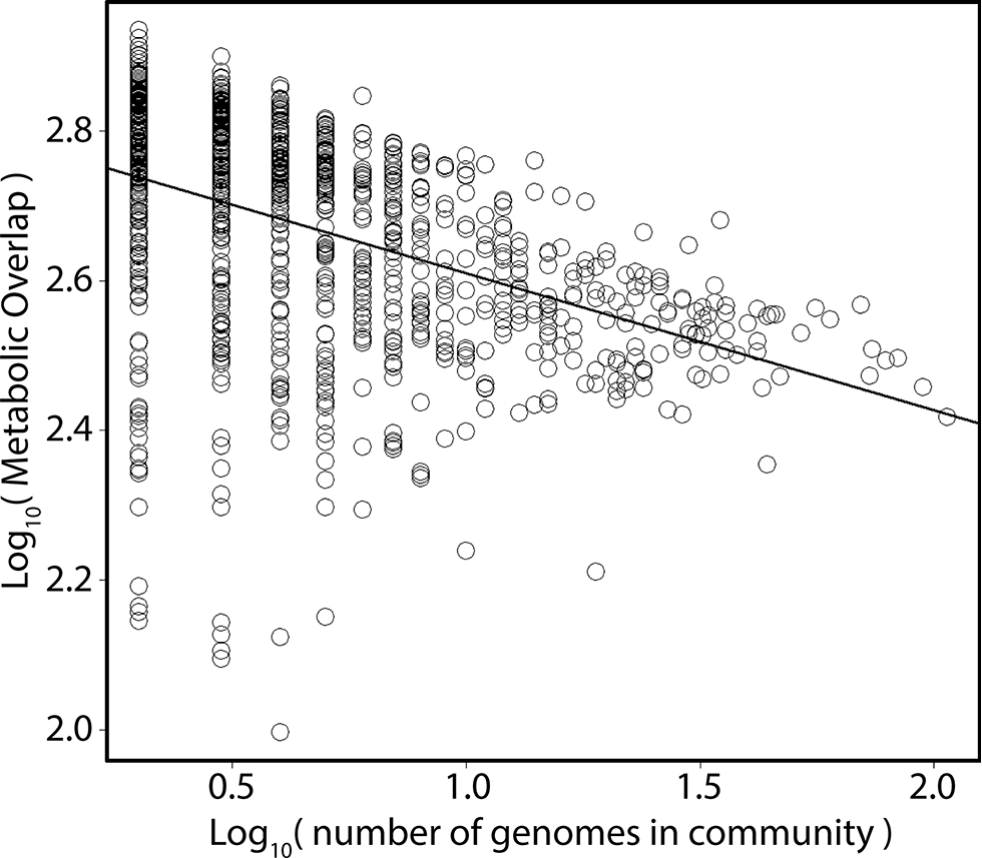
Relationship between metabolic overlap and the number of genomes in a community. Each point represents one of the 962 studies. The *x* axis depicts the total number of MAGs in a given study; the *y* axis, the mean metabolic overlap of that study.

We defined MO as the number of compounds (i.e., reactants) that can be utilized by two organisms based on their shared metabolic network (**Figure 1**). For example, an organism (Org_1_) that can perform all steps of denitrification from nitrate (NO_3_^−^) to nitrogen gas (N_2_, four reactions in total) shares two reactants with a partially denitrifying organism (Org_2_) that only reduces NO_2_^−^ to N_2_O. This then results in a MO  =  2 (ignoring the rest of their metabolism). To obtain a value that reflects the degree in which species in a community have overlapping niches, we calculated the median MO between all MAGs in a given study. These studies were grouped into distinct ecosystems based on their origin (**Figure 2**, **Table 1**). Conceivably, identifying MO allows a broad identification of species with overlapping niches by counting the compounds that link complementary metabolic pathways. As the metabolic routes used to degrade certain substrates can vary between organisms, counting the number of shared reactants will reveal MOs that would not be uncovered by shared reactions only. Furthermore, as the number of reactants can vary between reactions, this approach is more sensitive in identifying weak metabolic similarities between organisms.

**Table 1:** Number of studies and metagenomes within each ecosystem.

	Fresh water	Brackish	Extreme	Marine	Built	Animal	Engineered	Plant	Soil
Amino Acid	4.97E-05	4.24E-05	5.22E-05	4.63E-05	4.06E-05	3.33E-05	3.59E-05	4.09E-05	3.52E-05
Aromatic	6.61E-06	3.26E-06	6.55E-06	8.59E-06	6.91E-06	1.05E-06	2.80E-06	4.43E-06	6.02E-06
carbohydrates	5.58E-05	5.29E-05	6.03E-05	5.42E-05	5.09E-05	4.64E-05	4.33E-05	4.53E-05	4.31E-05
Cofactors	5.27E-05	4.71E-05	4.99E-05	4.79E-05	4.15E-05	3.20E-05	3.34E-05	4.12E-05	3.68E-05
Fatty acids	6.49E-05	7.01E-05	6.32E-05	6.13E-05	5.58E-05	5.33E-05	4.67E-05	5.07E-05	4.35E-05
Nitrogen	4.80E-06	4.90E-06	4.17E-06	3.71E-06	4.40E-06	2.02E-06	2.40E-06	2.63E-06	3.37E-06
Nuleoside	2.27E-05	1.82E-05	2.39E-05	2.28E-05	1.97E-05	2.46E-05	2.29E-05	1.86E-05	1.89E-05
Nuelotide sugars	5.01E-06	4.57E-06	5.38E-06	4.01E-06	3.78E-06	4.62E-06	4.51E-06	3.10E-06	4.43E-06
Phosphorus	4.62E-06	4.07E-06	2.91E-06	3.87E-06	3.50E-06	3.58E-06	3.24E-06	1.93E-06	3.05E-06
Protein	1.88E-05	1.75E-05	2.50E-05	1.62E-05	1.29E-05	1.82E-05	1.63E-05	1.66E-05	1.49E-05
Respiration	8.11E-06	8.48E-06	7.24E-06	7.12E-06	6.27E-06	2.98E-06	4.87E-06	4.74E-06	5.34E-06
Secondary Metabolism	2.40E-06	2.11E-06	3.93E-06	2.29E-06	1.80E-06	1.88E-06	1.95E-06	3.35E-06	2.28E-06
Sulfur	3.10E-06	2.88E-06	2.65E-06	3.26E-06	4.17E-06	9.78E-07	1.45E-06	9.84E-07	2.34E-06

We acknowledge that previous efforts to predict microbe–microbe interactions within microbial communities have been made with similar logic to the current approach. In particular, the NetCooperate software, utilizing the NetSeed framework, is a method to identify putative interactions in a community. It does so by using genome information to predict auxotrophies of the organisms present, based on the incompleteness of certain biosynthesis pathways leading to a dependency of the respective organism to external sources of the lacking metabolite (Carr and Borenstein, 2012; Levy et al., 2015). Thus, the NetSeed/NetCooperate approach predicts complementarity between species, which consequently occupy distinct niches, while the goal of our MO approach is to identify to what extent two species fill a common niche.

### Metabolic Overlap of Microbial Communities in Different Ecosystems

In order to survey the degree of MO in various ecosystems from around the globe, thereby identifying the degree in which microbial species within the community overlap in the niches they fill, the set of Uncultivated Bacteria and Archaea (UBA) MAGs published by Parks et al. (2017) was utilized. Contrasting to the naming scheme, this set contained some MAGs of cultured species also. The average predicted genome completeness of these MAGs ranged from 50% to 100%. A completion-based inclusion threshold of MAGs was found to have a negligible impact on the average MO of communities (**Supplemental Figure 1**). In contrast, the number of MAGs included drastically decreased as a result of a more stringent threshold on genome completeness, resulting in ecosystems poorly or not at all represented (**Supplemental Figure 2**). Several studies included in the UBA dataset included only one MAG and were excluded from our analyses. In total, 6,727 MAGs from the Parks et al. dataset, representing 962 studies, were included (**Table 1**). Studies were classified into their respective ecosystems of origin based on information included in the submission to the public repository or by manual curation if this information was insufficient. This resulted in nine ecosystem categories (**Table 1**). In total, the reaction space consisted of 1,386 unique compounds predicted to be utilized by the organisms represented by the current set of MAGs.

**Figure 3:**
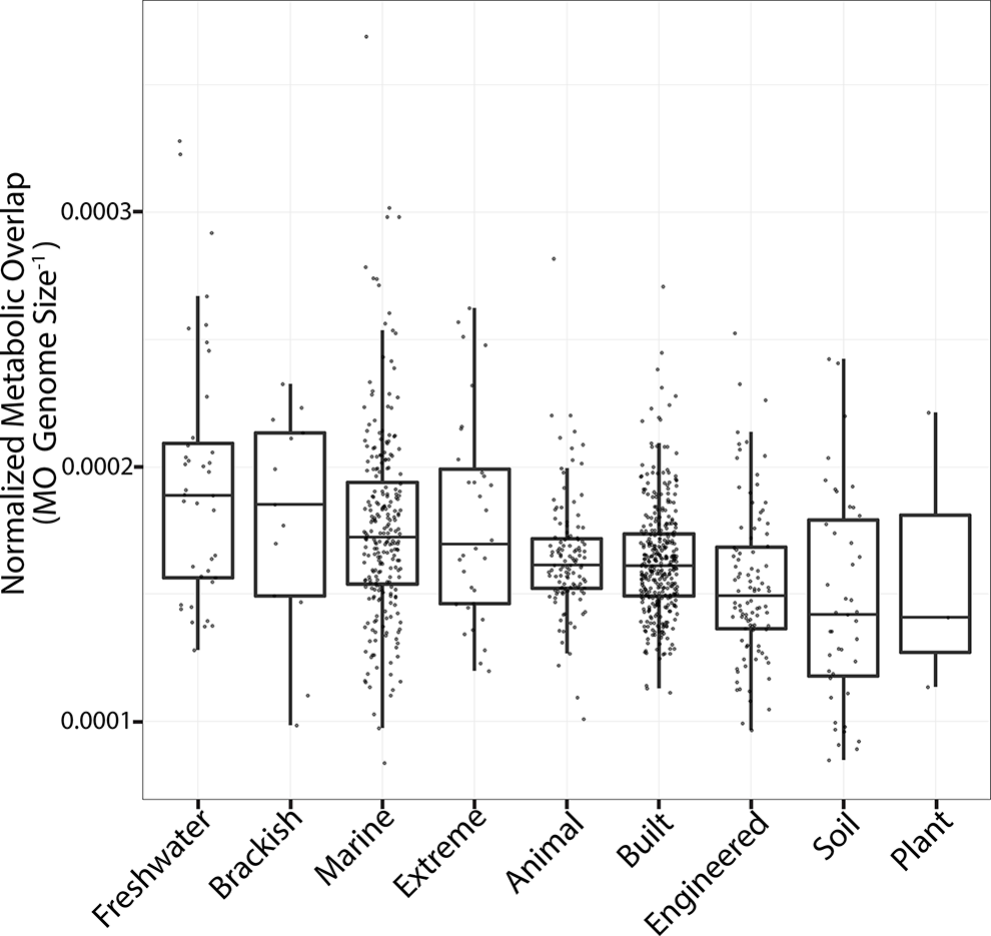
Metabolic overlap across all ecosystems. Boxplots are plotted with the black bar representing the median, the box corresponds to the 25% and 75% quartiles, and the whiskers are the extreme values. The *y* axis is MO normalized by genome size to account for differences between median genome sizes across ecosystems. The ecosystems are sorted from left to right based on the median MO. Each point represents the median MO of all MAGs from a given study.

In a given ecosystem, MO and the predicted average genome sizes of MAGs were strongly correlated (**Supplemental Figure 3**; *p* < 0.01). In addition, average genome sizes significantly varied between ecosystems (**Supplemental Figure 4**; ANOVA; *F*  =  88; *p* < 0.001). The average predicted genome sizes were the highest in studies from the built environment (4 ± 0.65 Mbp) and lowest in extreme environments (2 ± 0.96 Mbp). The number of MAGs in a given community (grouped per study) negatively correlated with the average MO of the community (**Figure 2**; Kendall τ =  −0.38; *p* < 0.001). As we were interested in investigating how MO varied between ecosystems, irrespective of the differences in genome sizes between ecosystems, we normalized MO to the median genome size of the respective study (**Figure 3**). MO was found to vary significantly between ecosystems (χ^2^  =  75.3; *p* < 0.001). Communities from animal, built, engineered, and soil ecosystems had significantly lower MO than aquatic ecosystems (*p* < 0.05; **Figure 3**, **Supplemental Table 1**). Furthermore, extreme ecosystems had significantly higher MO than built and engineered ecosystems (*p* < 0.05; **Figure 3**, **Supplemental Table 1**).

### Breakdown of MO Scores Across Different Ecosystems to Different Levels of Metabolism

To investigate how MO varied between ecosystems within different categories of metabolism (SEED subsystems), the MO within these subcategories was determined for each ecosystem and compared to the average value of all ecosystems (**Supplemental Table 2**). All metabolic subsystems varied between ecosystems (Kruskal–Wallis; *p* < 0.001; **Supplemental Table 2**). Animal, built, and engineered ecosystems in general had a lower MO for the majority of subcategories of metabolism with a few exceptions (Dunn; *p* < 0.01; **Supplemental Tables 3–15**). In contrast, communities from the engineered ecosystems had higher MO in protein and nucleotide sugar metabolism, as did communities from animal ecosystems. While most subcategories of metabolism from the built environment had lower MO than other ecosystems, these communities contained higher MO in nitrogen and sulfur metabolism (**Supplemental Table 16**). In contrast to the above communities, which were dominated by lower than average MO scores, extreme, freshwater, and marine ecosystems had higher than average MO scores in the majority of the categories of metabolism (**Supplemental Tables 3–15**).

**Figure 4:**
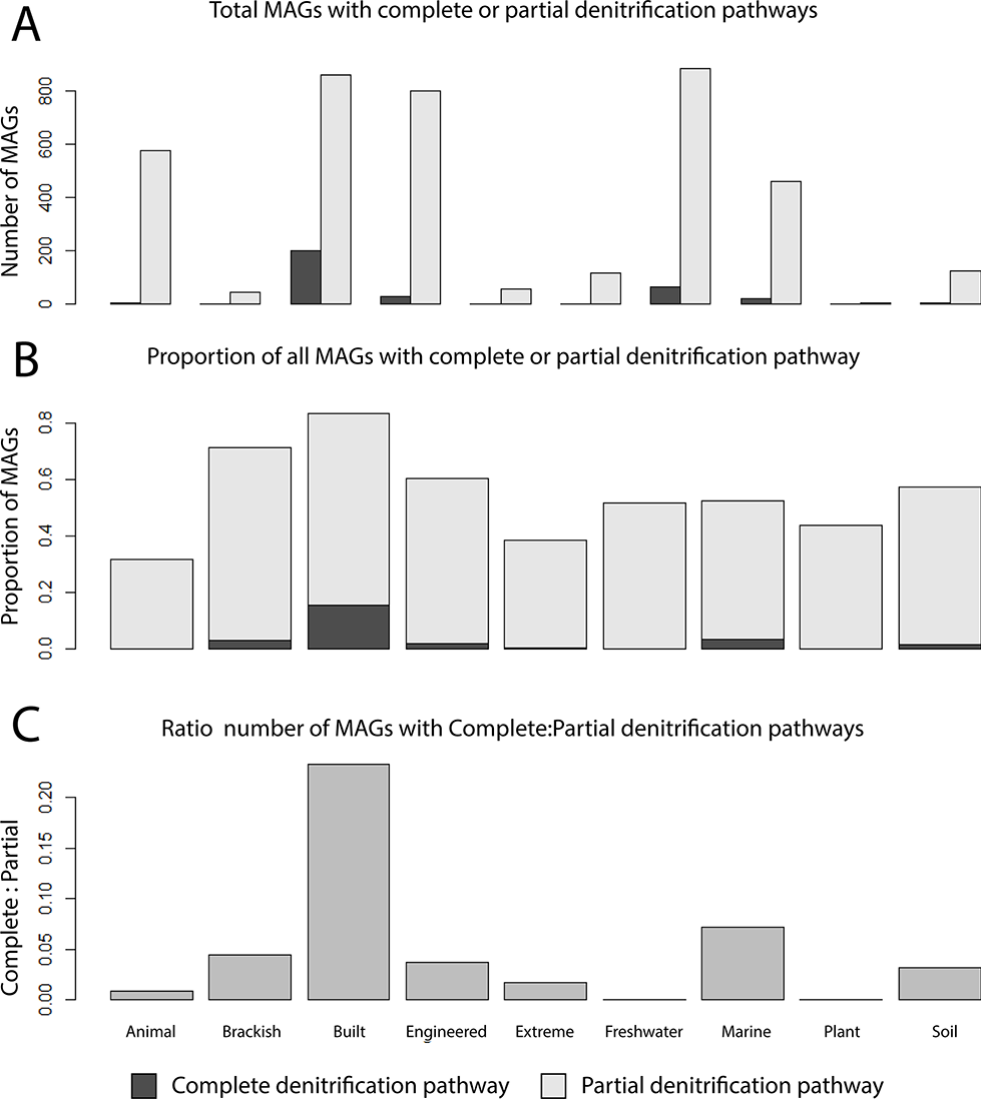
Proportions of complete and partial denitrifiers across different ecosystems. **(A)** Number of MAGs encoding all proteins to reduce NO_3_^−^ to N_2_ (complete denitrifiers) compared to the number of MAGs with one or more of the respective genes missing. **(B)** Proportion of MAGs of the total community that were either partial denitrifiers or complete denitrifiers. **(C)** Ratio of complete to partial denitrification pathways.

Nitrogen metabolism was used to further investigate the influence of partial pathways on the MO. Therefore, the ratios of complete to partial denitrifiers were calculated for all ecosystems (i.e., complete denitrifiers encoding all proteins required for NO_3_^−^, NO_2_^−^, NO, and N_2_O reduction; partial denitrifiers missing at least one gene; **Figure 4A**). The proportion of MAGs containing at least one denitrification gene ranged between ecosystems, with the lowest in the animal ecosystem and the highest in the built environment (**Figure 4B**). The built environment contained one of the highest MO in nitrogen metabolism and also had the highest ratio of complete to partial denitrifiers of all other ecosystems (**Figure 4C**). Contrary, the animal ecosystem, which by far had the lowest MO in this category, also contained mostly partial denitrifiers (**Figure 4C**).

### Phylogenetic Relationship of Organisms and Its Relationship to the MO

**Figure 5:**
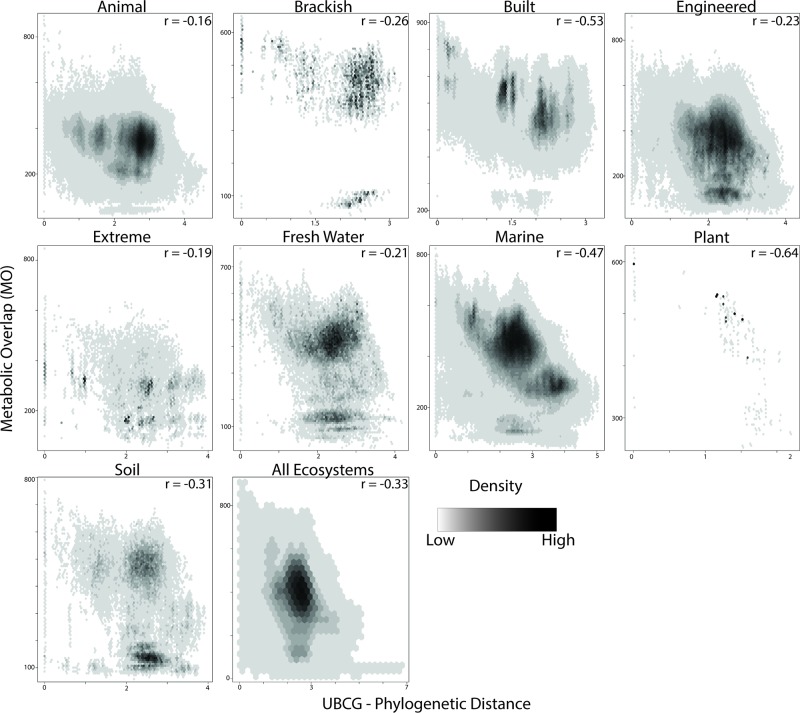
Relationship between metabolic overlap and phylogenetic distance. Each point represents a pairwise comparison between two MAGs. The density of points is represented by a black and white gradient. The Spearman correlation coefficient is indicated in the upper left-hand corner of each plot.

In order to determine if the evolutionary relatedness between MAGs was correlated with MO, the UBCG pipeline was utilized to infer a phylogenetic tree based on a concatenated alignment of 92 universal bacterial marker genes (Na et al., 2018). A significant negative correlation was observed between phylogenetic distance and MO for all ecosystems (**Figure 5**; *r*  =  −0.33; *p* < 0.001); however, the strength of this association varied. Phylogenetic distance and MO had the strongest association in plant (*r* = −0.64), built (*r*  =  −0.53) and marine ecosystems (*r*  =  −0.47), whereas the lowest associations were seen in animal (*r*  =  −0.16), extreme (*r*  =  −0.19) and freshwater ecosystems (*r*  =  −0.21; **Figure 5**).

## Discussion

In the current study, a new metric termed MO, which describes how similar two species’ metabolisms are, was developed in the context of a genome-based survey of microbial communities from diverse ecosystems. High MO between two species suggests that they have the capacity to perform similar metabolic reactions and thus have similar growth requirements and fill similar niches. In contrast, low MO suggests that the two species in question may compete for fewer resources. Thus, the average MO of a community can be interpreted such that in a community with high MO many community members are overlapping in their biochemistry and could in theory compete for a similar niche, whereas a low average MO would suggest the opposite.

### Ecological and Evolutionary Drivers of MO

There are several well-studied ecological forces that shape microbial community structure. Community diversity is maintained *via* dispersion (immigration and emigration) as well as speciation and extinction. In studying patterns of microbial biogeography, dispersion limitations were seen as one of the driving forces in structuring microbial community patterns in salt marshes and rice paddies and likely have an influence on the genomic adaptations of marine microorganisms (Martiny et al., 2006; Kelly et al., 2014; Lüke et al., 2014). Microbial biogeography theory has also been applied to help understanding compartmentalized host-associated microbial communities such as microbes in the human lungs (Whiteson et al., 2014). In this study, we observed major ecosystem-dependent differences in the MO of microbial community members (**Figure 3**). This variation may in part be attributed to dispersion limitations inherent to each ecosystem, where ecosystems in which the dispersion of microbial community members is limited would have less overlap than open homogenous ecosystems. Accordingly, the highest MO was observed in aquatic ecosystems, namely, communities from the marine open ocean environment, while animal host-associated communities contained some of the lowest MO (**Figure 3**). Ecosystems such as the ocean are likely to not have as strong dispersal limitations as ecosystems like the animal gut or human lungs, and these differences may be a driving force in structuring the MO of their respective microbial communities.

In addition to dispersion as an ecological force, disturbances to ecosystems can also play a large role for species diversity, driving extinction or speciation within the community (Connell, 1978; Buckling et al., 2000). Varying degrees of disruption would impart some signature on the metabolic pathways represented in the microbial community. A higher frequency of disturbance would contribute to the extinction of species and reduce the number of redundant metabolisms in a given system. For example, disturbances associated with the marine ecosystem (high MO) such as storms or temperature anomalies are likely less frequent and intense than the regular consumption of foodstuff or intermittent bouts of inflammation in animal guts (low MO) (Kashyap et al., 2013; David et al., 2014; Reese et al., 2018).

### Substrate Spectrum as a Possible Driver of MO in Ecosystems

The availability of resources, both in quality and quantity, drives which species can thrive in a given system. In the open ocean, the input of labile organic matter is a major factor controlling microbial activity in the photic zone, where phototrophs fix large quantities of inorganic carbon, making new organic matter available to heterotrophic organisms (Hansell and Carlson, 2002; Aylward et al., 2015). It is understood that differences in the composition of dissolved organic matter enrich for different clades of microorganisms and that the composition of the community is highly influential on the capacity to degrade this carbon (Nelson et al., 2013; Solden et al., 2018). In the case of animal- and plant-associated microorganisms, the composition of substrates provided to the microorganisms is often host-specific, which is thought to drive species specificity of the microbiota (Berg et al., 2014; Nelson et al., 2013; Hester et al., 2016; Quinlan et al., 2018; Reese et al., 2018; Jones et al., 2019). It would follow that a higher substrate selection would drive diversity in the microbial community, and the higher diversity of substrates would then lead to more diverse microbial metabolisms. In the current study, a negative relationship between the richness of a community (number of genomes in a given sample) and their average MO was observed, which suggests that in more diverse communities there is less MO (**Figure 2**).

In addition to the quality of substrates, the quantity of organic matter also drastically differs between ecosystems. The concentration of dissolved organic carbon (DOC) can vary greatly in aquatic systems, with around 40 µmol L^−1^ DOC in groundwater and 5,000 µmol L^−1^ in swamps and marshes (Søndergaard and Thomas, 2004). Likewise, variations in animal’s diet influence the availability of different substrates for microorganisms. In particular, the diet of an animal influences the availability of nitrogen to microbes in animal guts (Reese et al., 2018). Equally, N availability has a strong impact on plant-soil feedbacks, influencing the abundance and metabolism of microorganisms in the rhizosphere (Hester, 2018). If substrates are available in high-enough concentrations, the effect of competition may be reduced, potentially leading to a higher number of species consuming a common substrate (i.e., higher MO). In the current study, we observe microbial communities from animal ecosystems had the lowest overlap in categories of metabolism involved in nitrogen and amino acid metabolism, which corresponds to the idea of N limitations in the animal gut and known auxotrophies (**Supplemental Tables 3 and 8**; Reese et al., 2018; Zengler and Zaramela, 2018). In contrast, microbial communities from the built environment tend to have higher overlap in nitrogen and sulfur metabolism, although the built environment is a loosely defined ecosystem type with limited literature detailing nutrient fluxes through the system (**Supplemental Tables 8 and 15**; Adams et al., 2015). This stark contrast of nitrogen metabolism overlap between the built and animal ecosystems, which both generally displayed a lower than average MO, corresponded to the observed number of species capable of complete denitrification. The built ecosystem had the highest nitrogen metabolism MO, which largely was attributed to the highest proportion of microbial species capable of complete denitrification (**Figure 4**). This was contrasted by the low number of complete denitrifiers in the animal system. While the differences here could be due to nutrient availability, one should also consider possible differences in life strategies for persisting in a particular environment (i.e., detoxification vs. energy conservation).

### Influence of Phylogenetic Relationship on MO

Populations that become isolated and diverge on an evolutionary timescale do so as a result of being exposed to different environments and thus different selection pressures on specific traits, although some mechanisms exist that make this divergence less clear (i.e., convergent evolution, horizontal gene transfer, etc.). In the current study, a correlation was observed between the MO of species and their phylogenetic relationship (**Figure 5**), with a reduced MO in taxa that are more distantly related. While this corresponds well to theory, the strength of the relationship between phylogenetic relatedness and MO varied between ecosystems, suggesting that ecological differences between these ecosystems influence this relationship.

The dominant taxonomic groups often vary between different ecosystems as a result of the underlying nutrient profiles or physical properties of those ecosystems. This may be a result of stronger selection pressures in a given ecosystem for traits specific to a few select phylogenetic groups (i.e., methanogenesis, ammonia, and nitrite oxidation), as opposed to traits that are more widespread (i.e., denitrification). Phylogenetic groups may vary in the number of traits (i.e., some groups are more metabolically versatile than others, which often is also reflected in larger genome sizes within these groups), and MO is determined by the number of reactions a given pair of species share. For example, Zimmerman et al. (2013) found that a set of phylogenetically diverse bacteria and Archaea had the potential to produce a subset of three extracellular enzymes. The ability to produce these enzymes was nonrandomly distributed phylogenetically. It follows that ecosystems that have strong selection pressures for metabolically diverse phylogenetic groups would have a weaker relationship between the phylogenetic relatedness and MO. Interestingly, within each ecosystem type, there was a strong positive correlation between genome size and MO (**Supplemental Figure 3**), and the observed negative relationship of phylogenetic distance and MO seemed to be related to genome size (**Figure 5**). The built environment, which contained the largest genomes out of all ecosystems (**Supplemental Figure 4**), also had the strongest negative relationship between phylogenetic distance and MO (**Figure 5**). On the other hand, genomes from the animal ecosystem were the smallest and also showed the weakest relationship between MO and phylogenetic distance. It thus appears that both genome size (i.e., number of genes) and phylogenetic affiliation (closely related species sharing similar pathways) jointly influence MO between a given pair of species.

### Caveats and Limitations of Genetic Predictions of MO

The emergence of vast amounts of sequence data has allowed the assembly of genomes of microorganisms from fragmented DNA isolated from the environment. The degree of information in whole genomes compared to that from marker genes (both phylogenetic and metabolic) is likely to provide significant advances in our understanding of the genetic organization of microorganisms. In addition, knowing that a certain set of genomes were physically in the same sample is advantageous in addressing fundamental questions about the ecology and evolution of microbial communities in natural settings. Unfortunately, there are still significant limitations when dealing with MAGs. Specifically, the amount of information lost in the process of genome assembly and binning reduces our understanding of population-level genetic variation. It is still challenging to assemble genomes from organisms of low abundance, in particular when communities are complex (Cleary et al., 2015; Ayling et al., 2019). This narrows our view of genetic linkages between microorganisms toward the highly abundant and thus frequently observed species. However, these are mainly technological limitations, with solutions like long read sequencing becoming more widely available. Additionally, there is a significant lack of information about the environments in which samples were taken in the public archives. For instance, knowing the abundance of an organism in the community would significantly aid in inferring ecological interactions. The absence of such information limits what can be assessed with metrics such as MO and calls for an urgent need to provide as much metadata on samples as possible.

In addition to the technical limitations mentioned above, there are also limitations in methods such as MO, which rely heavily on accurate automated annotation of genetic elements in genomes. Specifically, database quality is a key driver in the accuracy of survey studies such as the one presented here. A major issue is the inability to assign functions to many genes, even in the genomes of the most well-studied microorganisms (35% hypothetical proteins in *Escherichia coli* genome; Ghatak et al., 2019). Apart from the limitations to automatic annotation methods, there are different levels of biology associated with niches that are not captured in genome-level information. These limitations include a lack of information of whether a gene is transcribed, whether the transcript is translated to a functional product, and ultimately variations in affinity and activity of this protein. The variation in transport efficiency and regulatory mechanisms certainly contributes to the competitive advantage of an organism and thus the niche this organism fills. These complexities are not easily derived from genomic information. Complementary techniques, such as transcriptomics, proteomics, and exometabolomics, could supplement the approach presented here by highlighting pathways that are expressed or translated under a given condition. Ideally, as emphasized by Bowers et al. (2017), in order to improve discovery-based approaches that rely on machine readable formats of public repositories, additional information should accompany MAG submissions. This set of information would not only help assess the quality of the genome but aid in associating the genetic information to the biology and ecology of the organism. Ideally, such information should include conditions of the environment from which the species’ genome was obtained (i.e., pH and temperature) and, if the species was cultivated, any physiological parameters that may have been measured (i.e., growth rate, substrate usage profile and affinities, etc.).

## Conclusions

The observation of variation in MO across different ecosystems begs several questions about the nature of microbial community metabolism. Specifically, what drives metabolic versatility in microbial communities? Are there generalizable rules that can be deduced? Survey-based studies enriched with additional information, such as those highlighted above, may shed additional light on important factors that drive MO. In addition, there is an urgent need to complement predictions based on the genetics of microorganisms with phenotypic data. Ultimately, understanding drivers of microbial community metabolism will lead to a better ability to predict and engineer microbial communities for industrial or conservational purposes.

## Methods

### Data Origin and Annotation of Ecosystems

Metagenome-assembled genomes utilized in the current study comprised the set published by Parks et al. (2017). The UBA MAGs were downloaded from the authors’ repository (https://data.ace.uq.edu.au/public/misc_downloads/uba_genomes/). The accompanying data from the UBA MAG set, including CheckM metrics of predicted genome completeness and size, were obtained from the publication (Parks et al., 2017). Each study in the UBA set of MAGs was manually sorted into a set of nine ecosystems.

### Metabolic Overlap Calculation

All MAGs were subsequently annotated using a custom pipeline based on the SEED API (Overbeek et al., 2005; Aziz et al., 2008). In brief, protein encoding genes (pegs) were called from the assemblies using svr_call_pegs (http://servers.nmpdr.org/sapling/server.cgi?pod=ServerScripts). Each of these proteins was then assigned to a figfam with svr_assign_using_figfams (our annotations can be found at: ericrhester.com/metabolicOverlap/annotations/results.tar.gz). The association of a protein to a biochemical reaction was then made with svr_roles_to_reactions. Custom script (rxn_expandinfo) associated reactions with compounds from the reaction database, which is found on the ModelSEED git repository (https://github.com/ModelSEED). Finally, the number of compounds shared between two organisms’ set of biochemical reactions is calculated to create a pairwise MO score, and an overlap matrix was constructed to store this information. This was made using the custom python scripts rxn_to_connections and lists_to_matrix, respectively (https://github.com/ericHester/metabolicOverlap). The overlap matrix represents the MO of all organisms within a single community and the average MO of all of these organisms is utilized in comparison in this study.

In addition to an overall MO score for a community, the above approach was used to calculate the MO of various subcategories of metabolism for the respective community. In addition to the above, an additional step was performed where pegs were assigned to their respective SEED subsystems and filtered with a custom script utilizing svr_roles_to_subsys. With pegs assigned to these metabolic categories, the above pipeline was used to identify reactions and compounds shared between pairs of organisms, subsequently resulting in an overlap matrix similar to that above. In this case, the overlap matrix stores the MO of the community pertaining to a specific category of metabolism. Matrices and accompanying data were further analyzed in R (R Core Team, 2016).

### Relating Phylogenetic Distances of Mags to Their MO Within Communities

In order to associate the phylogenetic distance of assembled genomes to their MO, the UBCG pipeline was utilized (Na et al., 2018). This pipeline extracts 92 conserved phylogenetic marker genes and builds multiple alignments for each gene. The resulting alignments are concatenated, and a maximum likelihood tree is inferred. This tree was imported into R utilizing the *ape* package, and distances were extracted from the tree object with the *cophenetic* function (Paradis et al., 2004). The result is a distance matrix containing phylogenetic distances between each pair of MAGs. Subsequently, this phylogenetic distance matrix and the overlap matrix storing MO scores were correlated using the *mantel.test* function from the ape package. The Spearman rank correlation coefficient was calculated for each ecosystem subset.

## Data Availability Statement

The Uncultured Bacterial and Archaeal (UBA) MAGs were downloaded from the author’s repository (https://data.ace.uq.edu.au/public/misc_downloads/uba_genomes/). The accompanying data from the UBA MAG set, including CheckM metrics of predicted genome completeness and size, was obtained from the publication (Parks et al., 2017).

## Author Contributions

EH, SL, CW, and MJ designed the study. EH performed the analysis and drafted the manuscript. EH, SL, CW, and MJ contributed to the editing of the manuscript.

## Conflict of Interest

The authors declare that the research was conducted in the absence of any commercial or financial relationships that could be construed as a potential conflict of interest.

## Supplementary Material

Supplemental Figure 1 Relationship between the genome completeness and the average metabolic overlap observed (colored lines, right axis). Each point represents the median MO of a study, which includes MAGs with a given genome completeness or higher.

Supplemental Figure 2 The number of MAGs included in the analysis at a given genome completeness cutoff.

Supplemental Figure 3 Relationship between metabolic overlap and genome size. Each point represents one study. The *y* axis indicates the median metabolic overlap of all MAGs in one study, and the median genome size for all MAGs in this study is on the *x* axis.

Supplemental Figure 4 Genome sizes across ecosystems. The black bar of the boxplot indicates the median, the box edge represents the upper and lower quartiles, whiskers denote extreme values, and individual points are outliers.

Supplemental Table 1 Multiple comparisons for differences in median MO for each ecosystem.

Supplemental Table 2 Kruskal–Wallis test statistics for differences in metabolic overlap for different categories of metabolisms grouped by SEED subsystems.

Supplemental Table 3 Dunn multiple comparisons evaluating the amino acid metabolism subsystem.

Supplemental Table 4 Dunn multiple comparisons evaluating the aromatic compound metabolism subsystem.

Supplemental Table 5 Dunn multiple comparisons evaluating the carbohydrate metabolism subsystem.

Supplemental Table 6 Dunn multiple comparisons evaluating the cofactors metabolism subsystem.

Supplemental Table 7 Dunn multiple comparisons evaluating the fatty acids metabolism subsystem.

Supplemental Table 8 Dunn multiple comparisons evaluating the nitrogen metabolism subsystem.

Supplemental Table 9 Dunn multiple comparisons evaluating the nucleoside metabolism subsystem.

Supplemental Table 10 Dunn multiple comparisons evaluating the nucleotide sugar metabolism subsystem.

Supplemental Table 11 Dunn multiple comparisons evaluating the phosphorus metabolism subsystem.

Supplemental Table 12 Dunn multiple comparisons evaluating the protein metabolism subsystem.

Supplemental Table 13 Dunn multiple comparisons evaluating the respiration subsystem.

Supplemental Table 14 Dunn multiple comparisons evaluating the secondary metabolism subsystem.

Supplemental Table 15 Dunn multiple comparisons evaluating the sulfur metabolism subsystem.

Supplemental Table 16 Summary of the median metabolic overlap for each subsystem of metabolism for all ecosystems.
