# Supplementary figures and images for: Metabolic Overlap in Environmentally Diverse Microbial Communities
Source: Front Genet. 2019 Oct 17;10:989. doi: 10.3389/fgene.2019.00989 (PMC6811665; doi:10.3389/fgene.2019.00989)

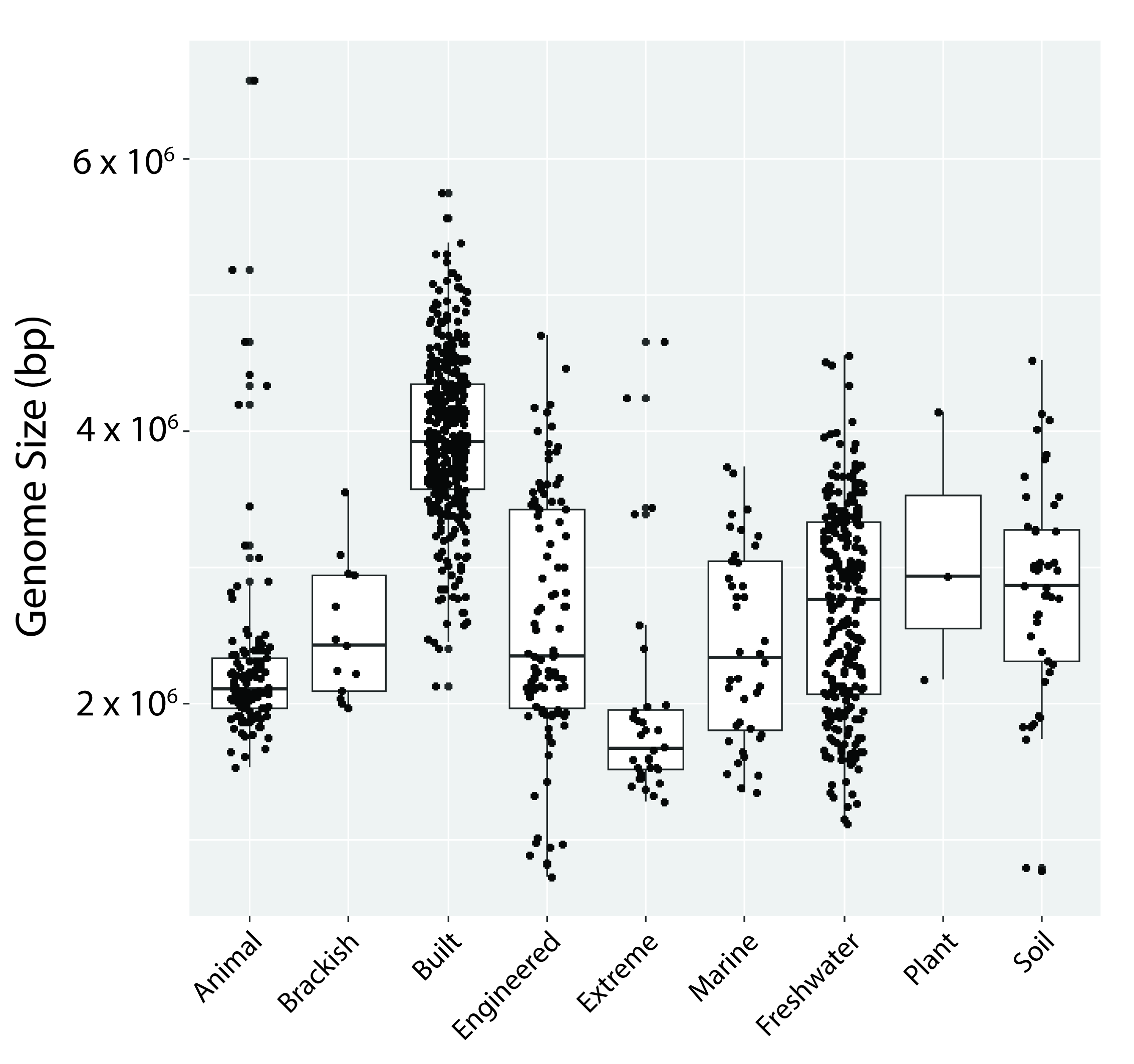

Supplement: Supplemental Figure 4 — Genome sizes across ecosystems. The black bar of the boxplot indicates the median, the box edge represents the upper and lower quartiles, whiskers denote extreme values, and individual points are outliers. [file Image_4.tif]
